# Nearly one in three children is suffering from sub-optimal feeding practice in Gibe District, Hadiya zone, South Ethiopia

**DOI:** 10.1186/s41043-020-00216-9

**Published:** 2020-11-09

**Authors:** Netsanet Fentahun, Yared Mulu, Tesfaye Feleke, Abraham Tamirat

**Affiliations:** 1 Department of Nutrition and Dietetics, School of Public Health, College of Medicine and Health Sciences, Bahir Dar University, Bahir Dar, Ethiopia; 2 School of Public Health, College of Medicine and Health Sciences, Bahir dar University, Bahir Dar, Ethiopia; 3 Department of Public Health, College of Medicine & Health Sciences, Arba Minch University, Arba Minch, Ethiopia; 4 Faculty of Public Health, Institute of Health, Jimma University, Jimma, Ethiopia

**Keywords:** Infant and young child feeding practice, Predictor, Hadiya zone, Ethiopia

## Abstract

**Background:**

Although infant and young child feeding practices play an important role, children in Ethiopia suffer from poor infant and young child feeding. To date, there is a limited study which addresses factors that influence infant and young child feeding practices. The aim of the study was to determine the predictors of infant and young child feeding practices in Gibe District, Hadiya Zone, Ethiopia.

**Methods:**

A community-based cross-sectional study was employed on 418 randomly selected mothers with children under the age of 24 months from March 13 to April 13, 2017. The pre-tested structured questionnaire was used to collect the data. Multiple logistic regressions were applied to determine the predictors of infant and young child feeding practices.

**Results:**

A total of 284 (67.9%) infant and young child suffered from the sub-optimal infant and young child feeding practices. The husband being a government employee [adjusted odds ratio (AOR) = 4.08 (1.65, 10.04)], lower household income [(AOR) = 3.11 (1.36, 7.07)], not attending antenatal care (AOR = 2.03 (1.22, 3.36)], child age 0–5 months [AOR = 2.42 (1.02, 5.72)], negative attitude towards infant and young child feeding practices [AOR = 2.35 (1.44, 3.84)], and the number of children 3–4 [AOR = 1.99 (1.08, 3.64)] were predictors of the sub-optimal infant and young child feeding practices.

**Conclusion:**

Sub-optimal infant and young child feeding practices were very high as compared to the WHO infant and child feeding recommendation. The husband being a government employee, lower household income, not attending antenatal care, child age 0–5 months, negative attitude towards infant and young child feeding practices, and the number of children 3–4 were the predictors of the sub-optimal infant and young child feeding practices. Nutritional interventions should emphasize the predictors of sub-optimal infant and young child feeding practices to improve optimal infant and young child feeding practices in Ethiopia.

## Introduction

The infant and young child feeding is a cornerstone of care for childhood development. The first 2 years of life provide a critical window of opportunity for ensuring children’s appropriate growth and development of children from generation to generation. Any damage caused during this period can lead to impaired cognitive development, compromised educational achievement, low economic productivity, and malnutrition [1–3].

WHO and UNICEF’s recommendations for optimal infant and young child feeding are early initiation of breastfeeding within 1 h of birth, exclusive breastfeeding for 6 months, and nutritionally adequate and safe complementary feeding starting from the age of 6 months with continued breastfeeding up to 2 years of age or beyond [1, 3, 4].

Globally, 156 million children were stunted, 50 million were wasted, and 42 million were overweight or obese. About 43% of infants are exclusively breastfed. Few children receive nutritionally adequate and safe complementary foods. In many countries, less than a fourth of infants 6–23 months of age meet the criteria of dietary diversity and feeding frequency that are appropriate for their age [5, 6].

Over 800 000 children can save their life every year, if all children 0–23 months were optimally breastfed. Improving child development and reducing health costs through breastfeeding results in economic gains for individual families as well as at the national level [6, 7].

Malnutrition has been responsible for 60% of the 10.9 million deaths annually among children under five. Two thirds of the deaths are associated with inappropriate feeding practices. Malnourished children are more frequently sick and suffer the life-long consequences of impaired development [6, 8]. Despite the reduction of undernutrition being one to achieve SDGs goal, it continues to be a great public health concern in many developing countries, particularly in sub-Saharan Africa [9, 10]. Millions of children globally suffer from undernutrition, despite many declarations and action plans aimed at combating the phenomenon [10, 11].

From EDHS 2016, only 14% of children age 6–23 months is fed appropriately, based on the recommended infant and young child feeding practices. It is also documented that poor infant feeding practices contribute to 24% of infant deaths [12, 13]. The aim of the study was to determine the predictors of infant and young child feeding practices in Gibe District, Hadiya Zone, Ethiopia.

## Methods

### Study area and period

The study was carried out in Gibe Woreda, Hadiya Zone, Southern Nation Nationalist of People Region, Ethiopia, which is located at 264 km from Addis Ababa (the capital city of Ethiopia), 230 km from Hawassa. The Gibe woreda is one of the highland areas in the country with an annual temperature estimated average minimum of 18 °C and maximum of 38 °C that can reach occasionally 45 °C and an annual rainfall of 1400 to 2000 mm. Gibe Woreda has a total population and households are 126,786 and 23,040, respectively. Among the total population of Woreda, 4186 are under 2-year children. There are 22 smallest administrative units in the district. There are one primary hospital, three health centers, and 22 health posts in the district. The study period was March 1–30, 2017.

### Study design and population

A community-based cross-sectional study design was conducted. Mothers with a child age less than 24 months in the randomly selected smallest administrative units were included in the study.

### Sample size and sampling techniques

The sample size was determined using a single population proportion formula by using the following assumptions: 44.7% received the minimum meal frequency [14], with 95% confidence level, marginal error of 5%, and non-response rate of 10%. Therefore, the final sample size was 418.

According to the World Health Organization recommendation for community-based study, we took eight out of 22 smallest administrative units. We used family registration books that had mothers with children age less than 24 months as a sampling frame. And then, the sample size was proportionally allocated to each smallest administrative unit. Finally, a simple random sampling technique was employed to select the study participants.

### Data collection instrument

The data were collected using pre-tested interviewer-administered questionnaire. It is adapted from previous studies and a WHO-recommended questionnaire for IYCF practices [1–3, and]. Socio-demographic factors were measured by using 15 items/questions. Maternal health service uses were assessed using 8 items with the yes/no and multiple-choice formats. The items were summed up to produce an index score; the higher score indicates more maternal health services utilization. Knowledge of mother was assessed using 42 items with the yes/no and choices. And then, we define as good and poor knowledge. Good knowledge of IYCF defines as mothers correctly answer 60% or above knowledge questions. Maternal attitudes towards IYCF practices were assessed using 25 Likert scale questions. A positive attitude about IYCF defines as mothers agree and strongly agree to favorable questions to appropriate IYCF.

The infant and young child feeding the index was derived as the summation of timely initiation of breastfeeding, exclusively breastfeeding, bottle feeding, timely introduction of solid, semi-solid and soft foods, minimum food diversification, and minimum meal frequency and continued breastfeeding. And then, the infant and young child feeding practice was classified as optimal feeding as a specific age group and score one, and sub-optimal and score zero. The scores were summed to generate an infant and young child feeding the composite index. Finally, the infant and young child feeding was dichotomized as an optimal infant and young child feeding for those having 4 or above, composite score [2].

#### Minimum dietary diversity

The proportion of children with 6–23 months of age who received foods from four or more food groups of the seven food groups [6, 15].

#### Minimum meal frequency

For children age 6–23 months who receive solid, semi-solid, or soft foods the minimum number twice for breastfed infants 6–8 months, three times for breastfed children 9–23 months, and four times for non-breastfed children 6–23 months [6, 15].

### Data collection methods and quality

Data were collected using an interviewer-administered questionnaire by using two local languages, Hadiya and Amharic. Two-degree and eight-diploma holders in health were recruited as supervisors and data collectors. The 1-day training was given on the objective, how they approach and interview the study participants. Supervisors were supervising and coordinating with their respective zone. Pretesting of the questionnaires was conducted on mothers who have infant 0–24 months, at Soro district, before the study period and appropriate modification was applied. All filled questionnaires were checked for completeness, accuracy, and consistency at end of each data collection day, and necessary corrections were made.

### Data processing and analysis

The data were edited, coded, and entered into EPI data version 3.1 and exported to SPSS version 21.0 statistical software for analysis. Further, data cleaning was made after exported to SPSS. Descriptive statistics were computed and presented using frequencies, proportions, summary statistics, graphs, and tables. Variables that have *P* value < 0.2 on bi-variate analyses were entered in the multivariable logistic regression model to identify independent predictors of IYCF. The strength of association and precision were examined using an adjusted odds ratio at a 95% confidence interval.

### Ethical consideration

Ethical approval was obtained from the Institutional Review Board (IRB) of the Jimma University Institute of Health. The formal supportive letter was obtained from the Department of Health, Behavior and Society. The necessary permission was obtained from the Hadiya Zone Health Department, SNNPR, Gibe woreda health office, and finally from health facilities. Verbal consent was obtained from the study participants. Participants were assured that their names will not be stated, and data will be kept confidential and anonymous. The participants were informed that this information was accessed by the investigator. They also informed that they were not forced to answer the entire question and they can withdraw at any time if they do not want to participate.

## Results

### Socio-demographic characteristics

A total of 418 of mothers and caregivers of children less than 24 months were included in the study. The mean age of the mothers was 30.72 years (SD ± 6.4) and ranges from 19 to 47 years. More than half 228 (54.5%) mothers were housewives and 111(26.6%) farmers. Regarding mothers’ educational status, 170 (40.7%) of mothers did not have formal education and 167 (40.0%) primary education. Most of the respondents were Hadiya by ethnicity (85.6%) and protestant by religion (70. 6%). More than half 211 (50.5%) of households had a family size of four to six, and the median family size was six. Regarding the wealth index, one to five (20.6%) of respondents were in 3rd quartiles. Regarding the husband education level, 205 (49.0%) and 101 (24.2%) of them had primary education and secondary education, respectively, whereas 245 (58.6%) of husbands were farmers by occupation (Table 1).

**Table 1:** Socio-demographic characteristics of respondents in Gibe District, Hadiya Zone, Southern Nation Nationalist of People Region, Ethiopia, 2017

Variables (*n* = 418)	Categories	Number	Percent
Age (years)	≤ 19	5	1.2
	20–29	197	47.1
	30–39	150	35.9
	≥ 40	66	15.8
Marital status	Married	403	96.4
	Widowed	9	2.2
	Divorced	6	1.4
Mother education	No education	170	40.7
	Primary education	167	40.0
	Secondary education	60	14.4
	Higher education	21	5.0
Mother occupation	House wife	228	54.5
	Farmer	111	26.6
	Private	62	14.8
	Government	17	4.1
Family size	1–3	47	11.2
	4–6	211	50.5
	≥ 7	160	38.3
Income	Lower	83	19.9
	Low	81	19.4
	Medium	86	20.6
	High	84	20.1
	Higher	84	20.1
Husband education (*n* = 403)	No education	38	9.1
	Primary education	205	49.0
	Secondary education	101	24.2
	Higher education	59	14.1
Husband occupation (*n* = 403)	Farmer	245	58.6
	Merchant	75	17.9
	Private	52	12.4
	Government	31	7.4

### Maternal and child characteristics

More than half of the 219 (52.4%) children were females and around one third of the 138 (33.0%) of them were 6–11 months old. Nearly one in three (65.8%) of the children was second to fourth in birth order. Around three–fourth of children (75.8%) birth intervals between the youngest children (index child) and his immediate older were less than 24 months (Table 2).

**Table 2:** Maternal and child characteristics in Gibe District, Hadiya Zone, Southern Nation Nationalist of People Region, Ethiopia, 2017

Variable (*n* = 418)	Number	Percent
Sex		
Male	199	47.6
Female	219	52.4
Child age (months)		
0–5	90	21.5
6–11	142	34.0
12–17	125	29.9
18–23	61	14.6
Birth order		
First born	48	11.5
2nd–4th	275	65.8
5th or more	95	22.7
Preceding birth interval (month)		
No previous birth	49	11.7
Less than 24	317	75.8
More or equal to 24	52	12.4
Antenatal care visit		
Yes	265	63.4
No	153	36.6
No of antenatal care visit		
1–2 times	148	35.5
3–4 times	87	20.8
> 4 times	11	2.6
Do not know	19	4.5
Place of delivery		
Home	270	64.6
Health institution	148	35.4
Mode of delivery		
Vaginally	395	94.7
Cesarean section	22	5.3
Post-natal care		
Yes	122	29.2
No	296	70.8
Number of children		
1–2	103	24.6
3–4	161	38.5
≥ 5	154	36.8
Parity		
Null-porous	49	11.7
Multi-porous	369	88.3

A total of 265 (63.4%) mothers attended antenatal care. One hundred forty-eight mothers (35.5%) had one to two antenatal care follow-up during their last pregnancy. Only 87 (20.8%) mothers attended three to four focused ANC as recommended. During the ANC follow-up, around three to four (76.4%) of the mothers did not receive information about IYCF. The majority of 396 (94.7%) of the mothers had a spontaneous vaginal delivery. Only 144 (34.4%) mothers gave birth with the assistance of skilled health professionals. A total of 296 (70.8%) of mothers did not attend the PNC. One hundred sixty-one (38.5%) mothers had three to four children. Most of the 369 (88.3%) of mothers were multi-porous with a mean of 4.2 live births (Table 2).

### Knowledge and attitude of mother’s on IYCF

Out of the total 418, 339 (81.1%) of the respondents had sufficient knowledge of IYCF practices. Out of 418 respondents, 189 (45.2%) had a positive attitude towards IYCF.

### Infant and young child feeding practices

**Fig. 1:**
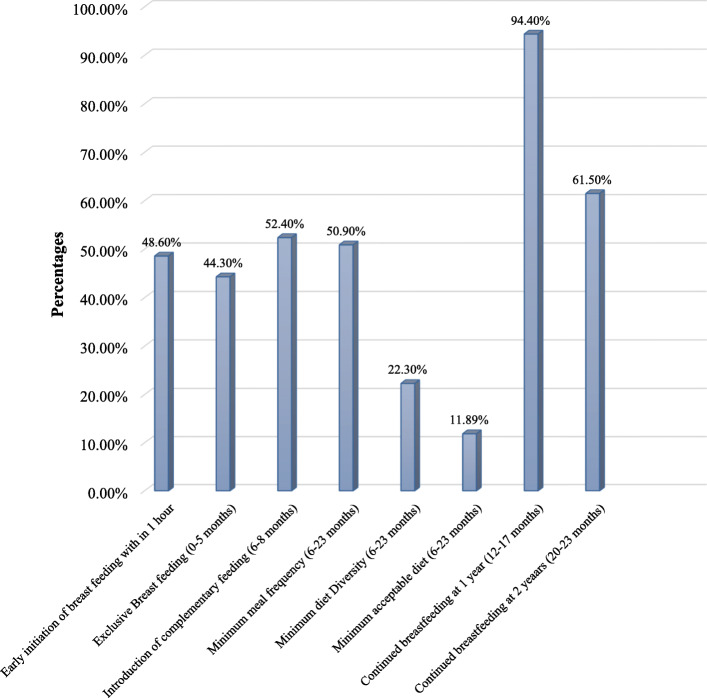
Infant and young child feeding practices from 0 to 23 months in Hadiya Zone, Gibe District, 2017

Figure 1 showed about infant and young child feeding practices. Almost all mothers 415 (99.3%) had ever breastfed their children. Less than half of the mothers of 203 (48.6%) initiated breastfeeding within the 1st hour of delivery. A total of 273 (65.3%) of the mothers gave colostrums to their infants. The study also showed that 207 (49.5%) mothers provided prelacteal foods for the baby. Overall, the prevalence of exclusive breastfeeding was 185 (44.30%). The mean age for the introduction of solid, semi-solid, and soft foods was 5.6 (SD ± 0.9) months.

A total of 172 (52.4%) was introduced as a complementary food at the age of 6–8 months. A total of 167 (50.9%) and 73 (22.3 %) received the recommended minimum meal frequency and dietary diversity, respectively. Only 39 (11.89%) infants and young children received the recommended minimum acceptable diet. The primary food source (99.1%) of children aged 6–23 months were grains, tubers, and roots, followed by dairy products (64.0%), whereas with the very smallest number (4.3%) of children were provided with fresh meat products. Only 39 (11.89%) received the recommended minimum acceptable diet. A total of 284 (67.9%) [95% CI; 63.6–72.5 infant and young children suffered from sub-optimal feeding practices (Fig. 1).

### Factors associated with IYCF

According to simple logistic regression analysis, mother occupation and education, husband occupation and education, family size, socioeconomic status, ANC, place of delivery, PNC, number of children, age of a child, birth order, and attitude of mothers toward IYCF were significantly associated with infant and young child feeding at *P* < 0.2. According to multiple logistic regression analyses, husband occupation, socioeconomic status, ANC, number of children, age of a child, and attitude of mothers were significantly associated with infant and young child feeding at *P* < 0.05.

Mothers who have a lower income 3.1 times more likely to practice the sub-optimal infant and young child feeding when compared with high income [AOR = 3.11 (1.36, 7.07)]. A government employee was 4.1 times more likely to practice the sub-optimal infant and young child feeding when compared with a merchant [AOR = 4.08 (1.65, 10.04). Infants in the age group 0–5 months were 2.4 times more likely to practice the sub-optimal infant and young child feeding when compared to those children with age group 18–23 months [2.42 (1.02, 5.72)].

Mothers who did not attend ANC visit were 2.1 times more likely to practice the sub-optimal infant and young child feeding when compared to those who attend the ANC visit [AOR = 2.1 (1.22, 3.36)]. Mothers who had a negative attitude towards IYCF were 2.4 times more likely to practice the sub-optimal infant and young child feeding when compared with those who have a positive attitude [AOR = 2.35 (1.43, 3.84)]. Mothers who had 3–4 children were 2 times more likely to practice sub-optimal infant and young child feeding compared with mothers who had 1–2 children [AOR = 1.99 (1.08, 3.64)] (Table 3).

**Table 3:** Multivariable logistic regression models predicting IYCF in Gibe District, Hadiya Zone, Southern Nation Nationalist of People Region, Ethiopia, 2017

IYC					
Variables		Inappropriate (%)	Appropriate (%)	COR(95% CI)	AOR(95% CI)
Mother education	Illiterate	140 (82.4)	30 (17.6)	3.29 (1.31, 8.31) ^*^	4.77 (0.97, 23.48)
	Primary	107 (64.1)	60 (35.9)	2.95 (1.17, 7.43) ^*^	4.16 (0.84, 20.55)
	Secondary	29 (48.3)	31 (51.7)	2.47 (0.89, 6.82)	2.58 (0.47, 13.97)
	Higher	7 (35)	14 (65)	1	1
Mother occupation	Government	6 (31.2)	11 (68.8)	1	1
	Private	27 (43.5)	35 (56.5)	1.70 (0.53, 5.47)	0.51 (0.05, 5.40)
	Farmer	81 (73)	30 (27)	5.94 (1.91, 18.52) ^*^	0.25 (0.02, 3.10)
	Housewife	170 (74.6)	58 (25.4)	6.45 (2.15, 19.34) ^*^	0.44 (0.04, 5.14)
Husband education	Illiterate	32 (84.2)	6 (15.8)	10.26 (3.42, 30.80) ^*^	0.81 (0.09, 7.50)
	Primary	160 (78)	45 (22)	6.84 (3.24, 14.44) ^*^	1.56 (0.32, 7.62)
	Secondary	59 (58.4)	42 (41.6)	2.70 (1.24, 5.88) ^*^	0.90 (0.22, 3.70)
	Higher	21 (35.6)	38 (64.4)	1	1
Husband occupation	Government	8 (25.8)	23 (74.2)	6.46 (2.88, 14.48) ^*^	**4.08 (1.65, 10.04)** ^*^
	Private	37 (71.2)	15 (28.8)	2.60 (1.32, 5.10)	1.60 (0.76, 3.41)
	Farmer	169 (73.5)	61 (26.5)	1.05 (0.58, 1.92)	4.52 (0.69, 29.55)
	Merchant	54 (72.0)	21 (28.0)	1	1
Family size	1–3	18 (38.3)	29 (61.7)	1	1
	4–6	139 (65.9)	72 (34.1)	0.15 (0.75, 0.31) ^*^	0.38 (0.02, 7.35)
	≥ 7	127 (79.4)	33 (20.6)	0.50 (0.31, 0.81) ^*^	0.58 (0.02, 17.07)
Monthly household income	≤ 500	38 (45.8)	45 (54.2)	2.50 (1.23, 5.04) ^*^	**3.11 (1.36, 7.07)** ^*^
	501–1000	56 (69.1)	25 (30.9)	0.81 (0.41, 1.59)	2.71 (0.66, 11.07)
	1001–1500	59 (68.6)	27 (31.4)	0.79 (0.40, 1.54)	2.18 (0.53, 9.01)
	1501–2000	69 (82.1)	15 (17.9)	1.65 (0.79, 3.48)	2.57 (0.78, 8.48)
	2000 & above	61 (72.6)	23 (27.4)	1	1
Age of child (month)	0–5	86 (95.6)	4 (4.4)	3.90 (1.81, 8.43) ^*^	**2.42 (1.02, 5.72)** ^*^
	6–11	95 (68.8)	43 (31.2)	1.65 (0.88, 3.08)	2.00 (0.67, 5.96)
	12–17	63 (50.4)	62 (49.6)	0.73 (0.39, 1.35)	0.76 (0.24, 2.40)
	18–23	36 (55.4)	29 (44.6)	1	1
Birth order	1–2	18 (36.7)	31 (63.3)	0.13 (0.07, 0.26) ^*^	0.18 (0.00, 8.09)
	3–4	127 (63.8)	72 (36.2)	0.40 (0.24, 0.64) ^*^	0.54 (0.05, 6.43)
	≥ 5	138 (81.7)	31 (18.3)	1	1
No of children	1–2	45 (43.7)	59 (57.3)	1	1
	3–4	115 (71.4)	46 (28.6)	1.89 (1.12, 3.20) ^*^	**1.99 (1.08, 3.64)** ^*^
	≥ 5	123 (80.4)	30 (19.6)	0.65 (0.36, 1.16) ^*^	0.77 (0.39, 1.49)
Antenatal care	Yes	150 (56.6)	115 (43.4)	1	1
	No	132 (86.3)	21 (13.7)	1.75 (1.15, 2.67) ^*^	**2.1 (1.22, 3.36)** ^*^
Place of delivery	HI	81 (54.7)	67 (45.3)	1	1
	Home	198 (73.3)	72 (26.7)	2.40 (1.57, 3.67) ^*^	4.35 (0.65, 2.83)
Post-natal care	Yes	74 (60.7)	48 (39.3)	1	1
	No	212 (71.6)	84 (28.4)	1.60 (1.03, 2.48) ^*^	0.76 (0.34, 1.72)
Attitude of mothers toward IYCF	Negative	168 (73.4)	61 (26.6)	3.25 (2.10, 5.00) ^*^	**2.35 (1.43, 3.84)** ^*^
	Positive	115 (60.8)	74 (39.2)	1	1

*Statistically significant at *p* value < 0.05, 95% CI

## Discussion

The aim of the study was to determine the predictors of the infant and young child feeding practices in Gibe District, Hadiya Zone, Ethiopia. The overall prevalence of the sub-optimal infant and young child feeding was 67.9%. WHO and UNICEF’s recommendations for optimal infant and young child feeding are early initiation of breastfeeding within 1 h of birth, exclusive breastfeeding for 6 months, and nutritionally adequate and safe complementary feeding starting from the age of 6 months with continued breastfeeding up to 2 years of age or beyond [1, 3, 4]. But the prevalence of the sub-optimal infant and young child feeding is very high in this study. The infant and young child feeding is a cornerstone of care for childhood development. The first 2 years of life provide a critical window of opportunity for ensuring children’s appropriate growth and development of children from generation to generation [1–3].

Around 21% of mothers fed their children using a bottle. WHO/UNICEF discourages the use of bottle feeding due to improper sanitation and formula preparation with bottle feeding can introduce microorganisms to the infant that increase the child’s risk of illness [16]. This finding is less than the national data and in Addis Ababa [15, 17]. Pre-lacteal feeding is one of the main factors contributing to under-five stunting [17].

Although global strategy on infant and young child feeding recommend feeding colostrums, in this study, 65.30% of mothers gave colostrums to their baby. This finding was lower than the study conducted in Mekelle town (82%) [18] and Arbaminch area (89.8%) [13]. There are many perceptions such as considered colostrum causes of diseases and abdominal pain which discourage the use of colostrum in the study area.

The prevalence of pre-lacteal feeding in this study is 49.7% which is much higher than the study done in Mekelle town, south Gondar zone and EDHS 2011 [13, 17, 18]. The prevalence of exclusive breastfeeding for infants less than 6 months was 44.3%. This result is lower when compared with the findings from the national targets, EDHS 2016, and Mekelle [15, 17, 19]. There are many common traditional practices which discourage the optimal infant and young child feeding practices such as introducing pre-lacteal feeding in the third day of birth for female, in the fourth day for males, and high home in the study area.

This study indicates that the diversity of different food groups offered during the past 24 h was low. Children in the 6–23 months of age group go through a reasonably rapid dietary transition from exclusive breastfeeding to complementary feeding. Additionally, during this dietary change, they are also prone to some diseases like diarrhea [20, 21]. During this period, children need more nutritious food to overcome the adverse effects of such diseases. Unfortunately, the current findings show that the children in this age group were not receiving appropriate complementary foods as recommended by the WHO. Our findings are similar to a national study and another study in the northern part of Ethiopia [12, 21].

Mothers who were lower income more likely to practice sub-optimal infant and young child feeding practice when compared with high income. This study similar to the study conducted in Saudi Arabia [22]. Likewise, the study conducted in Ethiopia showed that mothers who had low income were more likely to practice sub-optimal infant and young child feeding practice [15].

A government employee was 4.1 times more likely to practice the sub-optimal infant and young child feeding when compared with the merchant. This study is similar to the study conducted in Oromia Region. The possible reason might be merchants usually stay at home/around which help to give more attention to their child and have sufficient time, whereas government employee might not have adequate time give attention and obligated to their child early to go to work [23]. Mothers with infants in the age group 0–5 months were more likely to practice inappropriate IYCF as compared to those children with age group 18–23 months. This study is similar to the study conducted in Northwest Ethiopia [14].

Mothers who did not attend ANC visit were more likely to practice the sub-optimal infant and young child feeding practice when compared to those who attend ANC visits. This study is similar to the study conducted in Axum town, Ethiopia. Mothers who attend ANC with repeated visits have a great chance to get adequate information regarding infant and young child feeding practice [21].

Mothers who had a negative attitude towards IYCF were more likely to practice sub-optimal infant and young child feeding practice when compared with those who have a positive attitude. Women with 3–4 children were more likely to practice sub-optimal infant and young child feeding practice compared with women with children 1–2. This study is similar to the study conducted in Oromia Region, Ethiopia. Mothers who had a positive attitude towards the infant and young child feeding practice are more likely to adopt the optimal infant and young child feeding practice while counseling by health professionals [23]. The data collection was based on maternal recall, and mothers may have difficulty remembering some of the variables. So it might be subjected to the potential of recall bias, and it might overestimate or underestimate the result. Even though optimal infant and young child feeding practices have a direct effect on child nutrition and development, this study did not link with the optimal infant and young child feeding practices. In addition, the maternal nutrition was not assessed in this study.

### Public health significance

The optimal infant and young child feeding practices is an effective intervention to improve child nutrition and reduce child mortality in developing countries. In this study, the finding shows that infants are suffering from sub-optimal infant and young child feeding practices. This finding has policy implications on how to address the sub-optimal infant and young child feeding practices problem in developing countries like Ethiopia. Additionally, this finding influences our understanding of the sub-optimal infant and young child feeding practices intervention dimensions. And sub-optimal infant and young child feeding practices have an impact on the design of target-oriented breastfeeding promotion interventions.

## Conclusions

Sub-optimal infant and young child feeding practices were very high as compared to the WHO infant and child feeding recommendation. The husband being a government employee, lower household income, not attending antenatal care, child age 0–5 months, negative attitude towards the infant and young child feeding practices, and the number of children 3–4 were predictors of the sub-optimal infant and young child feeding practices. Nutrition interventions should emphasize the predictors of sub-optimal infant and young child feeding practices to improve optimal infant and young child feeding practices in Ethiopia.

## Data Availability

The datasets supporting the conclusions of this article are included within the article.
